# Chemical Composition of Wild Fallow Deer (*Dama Dama*) Meat from South Africa: A Preliminary Evaluation †

**DOI:** 10.3390/foods9050598

**Published:** 2020-05-07

**Authors:** Donna-Mareè Cawthorn, Leon Brett Fitzhenry, Radim Kotrba, Daniel Bureš, Louwrens C. Hoffman

**Affiliations:** 1 Department of Animal Sciences, Faculty of AgriSciences, University of Stellenbosch, Private Bag X1, Matieland 7600, South Africa; donna.cawthorn@ump.ac.za (D.-M.C.); lbfitzhenry@gmail.com (L.B.F.); 2 School of Biology and Environmental Sciences, Faculty of Agriculture and Natural Sciences, University of Mpumalanga, Cnr R40 and D725 Roads, Nelspruit 1200, South Africa; 3 Department of Animal Science and Food Processing, Faculty of Tropical AgriSciences, Czech University of Life Sciences Prague, 165 00 Prague 6-Suchdol, Czech Republic; maugli46@volny.cz; 4 Department of Ethology, Institute of Animal Science, 104 00 Prague 10-Uhříněves, Czech Republic; 5 Department of Food Science, Faculty of Agrobiology, Food and Natural Resources, Czech University of Life Sciences Prague, 165 00 Prague 6-Suchdol, Czech Republic; bures.daniel@vuzv.cz; 6 Department of Cattle Breeding, Institute of Animal Science, 104 00 Prague 10-Uhříněves, Czech Republic; 7 Centre for Nutrition and Food Sciences, Queensland Alliance for Agriculture and Food Innovation, The University of Queensland, Coopers Plains, QLD 4108, Australia

**Keywords:** fat, moisture, minerals, protein, proximate composition, venison

## Abstract

Although fallow deer are abundant in South Africa, these cervids remain undervalued as a domestic protein source and little information exists on their meat quality. This study aimed to evaluate the proximate and mineral compositions of the meat from wild fallow deer (*n* = 6 male, n = 6 female) harvested in South Africa, as affected by sex and muscle. Proximate analyses were conducted on six muscles (*longissimus thoracis et lumborum* [LTL], *biceps femoris* [BF], *semimembranosus* [SM], *semitendinosus* [ST], *infraspinatus* [IS], *supraspinatus* [SS]), whereas mineral analyses were conducted on the LTL and BF. The proximate composition of the muscles ranged from 73.3–76.2% moisture, 20.4–23.1% protein, 2.2–3.2% fat, and 1.1–1.5% ash. Proximate composition was significantly (*p* ≤ 0.05) influenced by muscle, but not by sex. The primary essential macro- and micro-minerals determined in the LTL and BF were potassium, phosphorus, sodium, and magnesium, as well as iron, zinc, and copper, with more variation in concentrations occurring with muscle than with sex. Minerals in the muscles contributing most notably to human recommended dietary requirements were potassium, iron, copper, and zinc. These findings indicate that wild fallow deer meat is a nutritious food source and should enhance utilisation of such products.

## 1. Introduction

In many developing countries, growing human populations, higher incomes, and rapid urbanisation are spurring an unprecedented demand for foods of animal origin [1]. South Africa is evidently no exception, showing a ca. 60% rise in per capita meat consumption over the past 20 years and necessitating a shift towards more intensive production and increased meat importation to satisfy these escalating protein requirements [2,3]. Within the next 30 years, it is projected that the South African population will reach 73 million and that urbanisation rates will approach 80% [4,5]. Accordingly, meat supply will likely need to double or even treble to maintain pace, posing significant challenges in the face of growing natural resource scarcity and climate change [4]. For one, the amount of land available for livestock farming is being continually reduced to make way for human settlements and other agricultural or extractive industries. Much of the land that remains is degraded, primarily as a result of overgrazing by domestic livestock [3]. Moreover, recurrent droughts are further draining water supplies and exacerbating animal feed shortages, leading to livestock deaths and/or forced downscaling of herd sizes [6]. The joint effects of these limitations, compounded by increasing carbon constraints and tightening environmental and animal welfare legislation, could cause meat yields to fall considerably short of anticipated demands. Such a realisation would likely present a major impediment to achieving food security in South Africa, where chronic hunger, protein malnutrition, and micronutrient deficiencies are already pronounced [4,7].

Given the restrictions on improving the productivity of domestic livestock, emphasis is progressively shifting to the role of alternative species in providing high-quality protein for human consumption. More specifically, there is growing recognition of the importance of utilising marginal and sub-marginal lands more optimally by harvesting or stocking species that thrive under adverse conditions, especially those that are abundant in the wild or are considered as pests [8]. Such an endeavour is epitomised by the game ranching industry in South Africa, whereby the stocking of wild ungulates that are adapted to local conditions allows ranchers to operate with lower input costs compared with livestock farming, while simultaneously reducing land degradation, limiting carbon emissions, and maintaining biodiversity [9]. This industry not only provides a valuable source of meat for consumption, but has also directly contributed to a 40-fold increase in game numbers in the country over the last 50 years [10]. The local retail and export market for game meat generates ca. ZAR 230 million (15–19 million tonnes) per annum, with springbok (*Antidorcas marsupialis*), kudu (*Tragelaphus strepsiceros*), blesbok (*Damaliscus pygargus phillipsi*), impala (*Aepyceros melampus*), gemsbok (*Oryx gazella*), and wildebeest (*Connochaetes* spp.) contributing most notably [11]. All these animals come from free-range, extensive production systems.

One wild ungulate species in South Africa that has received far less attention as a meat producer is the fallow deer (*Dama dama*), which was introduced into the country by the British in the 19th century [12]. Although fallow deer have adapted well to South African conditions, have proliferated in number, and are now often regarded as agricultural pests [13], these animals currently contribute minimally to the formal game meat industry and to local food security. In contrast, fallow deer are widely harvested and/or farmed in Europe, North America, and Oceania, where their meat (venison) is a highly marketed commodity [14]. One reason for the slow uptake of fallow deer meat in South Africa likely lies with the lack of information on its chemical composition and nutritional value, which in turn translates to a poor understanding of the quality and potential health benefits of such products.

Like the meat from African antelope, deer meat is typically high in protein (>20%), low in intramuscular fat (IMF; <3%), and rich in micronutrients [15]. Nonetheless, it is known that the chemical composition of muscle tissue can be influenced by a range of intrinsic and extrinsic factors, with the IMF content generally being the most variable [16]. Compositional differences between animals within a species are mostly attributed to age, sex, and diet. For instance, the levels of IMF tend to be higher in meat from older animals, as do the levels of certain minerals [17]. Moreover, the females of most ungulate species are inclined to have higher IMF reserves than the males [18], which helps to improve their chances of reproductive success and buffer the energetic costs of gestation [19]. Male ungulates, on the other hand, generally lose substantial body fat during the rutting and mating season when feeding is reduced and considerable energy is expended on fighting for dominance and maintaining the harem [20,21]. Additionally, IMF concentrations can vary considerably between muscle types within an individual animal, largely due to differences in the number and size of intramuscular adipocytes between muscle fibres, but also owing to differences in the muscle fibre types themselves [17,22]. At present, the impacts of the aforementioned factors on the composition of South African fallow deer meat remain unquantified and such an understanding will be imperative if the meat industry is to deliver consistent and desirable products to the consumer.

The aim of this study was, thus, to generate baseline data on the proximate and mineral composition of meat from wild fallow deer harvested in South Africa, as affected by sex and muscle. Furthermore, by comparing the chemical meat quality of local fallow deer with that of other deer, African game species, and domestic livestock, the objective was to establish whether the former can compete and serve as a complementary meat source for a largely protein-deficient nation.

## 2. Materials and Methods 

### 2.1. Harvesting and Slaughtering 

Ethical clearance for this study was granted by the Stellenbosch University Animal Care and Use Committee before commencement of field work (No.: SU-ACUM000-44). A full account of the methods used for harvesting and slaughtering of the fallow deer is detailed in Cawthorn et al. [23]. Briefly, 12 fallow deer (*n* = 6 males; *n* = 6 females) were night harvested (spotlight cropping) by licensed hunters in August (winter) on Brakkekuil farm (34°17’47.6"S; 20°49’28.0"E) near Witsand in the Western Cape province of South Africa. Although harvested on a farm, these animals were all from wild populations and had freedom to roam between farms in the region. As fallow deer are considered invasive in South Africa, there is no specific hunting season for these animals. Cropping was therefore performed for the purpose of controlling the fallow deer population size on the farm. 

All animals were shot with a single shot to the head, followed by immediate exsanguination in the field. The carcasses were subsequently tagged with unique identification numbers and transported to an on-site abattoir where individual slaughter weights were documented (Mettler Toledo Hawk Scale, supplied by Microsep, South Africa) within 2 h post mortem. After skinning and evisceration, the dressed carcasses were suspended by both Achilles tendons in a cool room (± 4 °C) for a period of 16 h. Additional information on the carcass’ characteristics is provided in Supplementary Table S1. 

Although adult fallow deer were targeted for this study, the specific ages of the animals were not estimated according to tooth wear as no baseline data exist for fallow deer tooth wear in South Africa. It should, however, be noted that hunting pressure by surrounding farmers was observed to have reduced the number of mature fallow deer buck in the study area. The slaughter weights of the harvested males and females were similar, with mean (± standard error) values of 43.5 ± 2.63 kg and 43.0 ± 3.19 kg, respectively (Supplementary Table S1). In comparison, mean body mass values of mature fallow deer bucks and does from the literature are 67 kg and 44 kg, respectively [24].

### 2.2. Muscle Removal and Sample Preparation 

Six muscles were excised from the right-hand side of each animal: *longissimus thoracis et lumborum* (LTL), *biceps femoris,* (BF), *semimembranosus* (SM), *semitendinosus* (ST), *infraspinatus* (IS), and *supraspinatus* (SS). The excised muscles were weighed separately (Digi DS-673 scale, B & R Scale Services, Cape Town, South Africa; Supplementary Table S1) and then individually vacuum packed (Multivac C200, Multivac, Gauteng, South Africa) and refrigerated (± 4 °C) overnight. The muscle samples were subsequently transported under chilled conditions to the laboratory at Stellenbosch University (South Africa). 

Each muscle was individually homogenised using a Dampa CT-35N Bowl Cutter (Mason Gray Strange, Kilkenny, South Australia). The homogenised content was subsequently divided, vacuum packed (Multivac C200), labelled, and stored at −20 °C. Prior to analyses, the homogenised samples were thawed (±4 °C, 24 h), with thorough mixing of each muscle homogenate to incorporate exuded moisture.

### 2.3. Proximate Analysis

The moisture contents (% wet weight) of 2.5 g homogenised meat samples were analysed in duplicate for all six muscles by drying for 24 h at 100 °C, following official method 934.01 of the Association of Official Analytical Chemists [19].

The total crude protein contents of the dried, defatted, and ground meat samples were determined in duplicate following the AOAC 992.15 Dumas combustion method [25]. Sub-samples (0.1 g) from each muscle homogenate were encapsulated in a Leco^TM^ foil sheet and were subsequently analysed in a Leco Nitrogen/Protein analyser (FP—528, Leco Corporation, St. Joseph, MI, USA). The Leco analyser was calibrated with ethylene-diamine-tetra-acetic acid (EDTA) (Leco Corporation) prior to analysis of each sample batch. The accuracy and recovery rate of the method was ensured by running a calibration sample of known protein content after every 10 test samples. The results obtained as % nitrogen (N) were multiplied by a conversion factor of 6.25 to determine total crude protein (%) values.

The IMF contents (% wet weight) of 5 g homogenised muscle samples were analysed in duplicate by means of a chloroform-methanol extraction gravimetric method [26]. A 1:2 (*v*/*v*) chloroform/methanol solution was used for extraction as the samples were expected to contain <5% fat.

The ash content (% wet weight) of dried meat samples (from the moisture analysis described above) was determined in duplicate by ashing at 500 °C for 6 h, following AOAC 942.05 [25]. 

### 2.4. Minerals

Mineral analyses were performed on samples of raw muscle homogenates from the LTL and BF of each fallow deer. Two muscles were selected for these analyses due to budgetary constraints and the considerable costs of such analyses. From each sample, 0.5 g was digested on a MARS 240/50 microwave digester (CEM Corporation, Mathews, North Carolina) using 6.5 mL ultra-pure nitric acid (HNO_3_) and 0.5 mL hydrochloric acid (HCl) (Merck Suprapur®) at elevated pressure (800 psi) and temperature (200 °C) in order to solubilise the acid-extractable elemental content. After cooling, the extracts were made up to 50 mL with deionised water in acid-cleaned Falcon tubes and were subsequently analysed for 26 elements. Major elements (calcium, magnesium, phosphorus, potassium, and sodium) were analysed on a Thermo ICap 6200 inductively coupled plasma atomic emission spectroscopy (ICP-AES) instrument (Thermo Fisher Scientific, Waltham, MA, USA) after calibration with NIST-traceable standards (catalogue no. IV-28, supplied by Inorganic Ventures Inc., Christiansburg, VA, USA) and validation using a multi-element standard (catalogue no. 1105800100, supplied by Merck Millipore, Darmstadt, Germany). 

Trace elements (aluminium, antimony, arsenic, barium, boron, cadmium, chromium, cobalt, copper, iron, lead, manganese, mercury, molybdenum, nickel, selenium (Se), silicon, strontium, titanium, vanadium, and zinc) were analysed on an Agilent 7700 quadrupole inductively coupled plasma mass spectrometry (ICP-MS) instrument (Agilent Technologies, Santa Clara, CA, USA). The instrument was tuned to optimise sensitivity and minimise oxides (<1% CeO/Ce ratio). Analysis was done using the Agilent-patented HMI functionality to minimise matrix effects and drift using helium (He) as collision cell gas for interference removal. Similar calibration (NIST-traceable standards, catalogue no. IV-28, Inorganic Ventures) and validation (multi-element standard, catalogue no. 1105800100, Merck Millipore) procedures were performed as for ICP-AES. Results were corrected to account for the dilution factors resulting from the digestion procedure, being expressed as mg/kg meat on a wet-weight basis.

### 2.5. Statistical Analysis

Statistical analyses were performed using Statistica 12 (www.statsoft.com). Linear mixed model repeated measures Analysis of Variance (ANOVA) was employed as measurements were conducted on different muscles (i.e., repeated measurements) from the same animal. Sex, muscle, and sex by muscle interaction (sex × muscle) were treated as fixed effects, while the animals were treated as a random effect. For post hoc analysis, Fisher least significant difference (LSD) tests were utilised. Where applicable, Pearson’s correlations were calculated for the various parameters. Differences were considered significant at 5% (*p* ≤ 0.05).

### 2.6. Comparative Data for Deer, Antelope, and Livestock

Comparative compositional data for South African antelope and ruminant livestock, as well as deer from abroad, were obtained by searching published and peer-reviewed literature indexed in academic bibliographic databases (Google Scholar, Science Direct, Scopus) and published dissertations in the “grey literature”, using the following two Boolean search strings: (i) (South Africa) AND (antelope OR bovid* OR cattle OR beef OR sheep OR mutton OR goat OR chevon) AND (proximate OR chemical OR nutrition* OR protein OR fat OR lipid OR mineral*) AND (longissimus OR loin); and (ii) (deer OR cervid*) AND (proximate OR chemical OR nutrition* OR protein OR fat OR lipid OR mineral*) AND (longissimus OR loin). The captured information was screened for relevance and filtered to retain only applicable sources. Data from the retained literature were collated into a central database and then plotted against the values derived through the current study.

## 3. Results 

### 3.1. Proximate Composition

**Figure 1:**
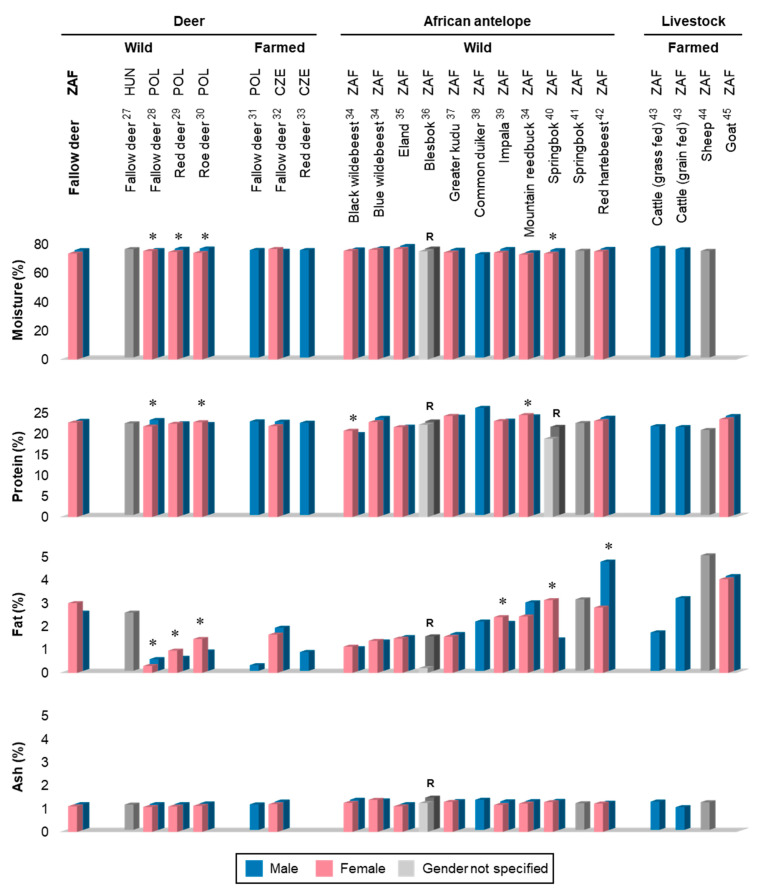
Proximate composition of *longissimus thoracis et lumborum* muscles from selected game and ruminant livestock species, including sex comparisons where applicable. Values obtained in the present study appear in the first column (label marked in bold). The superscript numbers indicated after each species refer to references in the reference list [27,28,29,30,31,32,33,34,35,36,37,38,39,40,41,42,43,44,45]. * Indicates studies in which a significant sex effect was found; “R” indicates studies in which a range of values were reported. Abbreviations: CZE = Czech Republic; DEU = Germany; HUN = Hungary; POL = Poland; ZAF = South Africa.

The proximate composition (g/100 g (%)) of the six muscles from male and female fallow deer is presented in Table 1, while the values obtained for the fallow deer LTL are compared with those of deer species from abroad, indigenous game, and domestic ruminant livestock in Figure 1 (see also Supplementary Table S2). 

No significant interactions were observed between the main effects (sex × muscle) in terms of proximate composition (Supplementary Table S3). When considering the main effects separately, sex did not have a significant influence on the levels of any of the proximate components measured in the six fallow deer muscles (Table 1). Conversely, the effect of muscle was significant in terms of all the proximate components measured. The moisture content was significantly higher in the SS, IS, and ST and lower in the SM and LTL. In terms of protein, significantly higher levels were found in the SM and LTL, while the lowest levels were found in the SS (Table 1). A negative correlation between protein and moisture values was calculated for all fallow deer muscles, namely LTL (*r* = −0.49), BF (*r* = −0.62), SM (*r* = −0.84), ST (*r* = −0.77), IS (*r* = −0.58), and SS (*r* = −0.57). The ratio between protein and moisture ranged from 0.27:1 to 0.31:1 in the various muscles. The IMF contents of the LTL, SM, ST, and SS did not differ significantly from one another, whereas the IS had the lowest IMF content (Table 1). A negative correlation between lipid and moisture was calculated for the LTL (*r* = −0.47), SM (*r* = −0.66), IS (*r* = −0.29), and SS (*r* = −0.24), but little correlation between these components was determined for the ST (*r* = −0.03) and BF (*r* = 0.03). In terms of ash, the highest levels were observed in the ST and the lowest in the LTL and BF (Table 1). 

### 3.2. Mineral Composition

**Figure 2:**
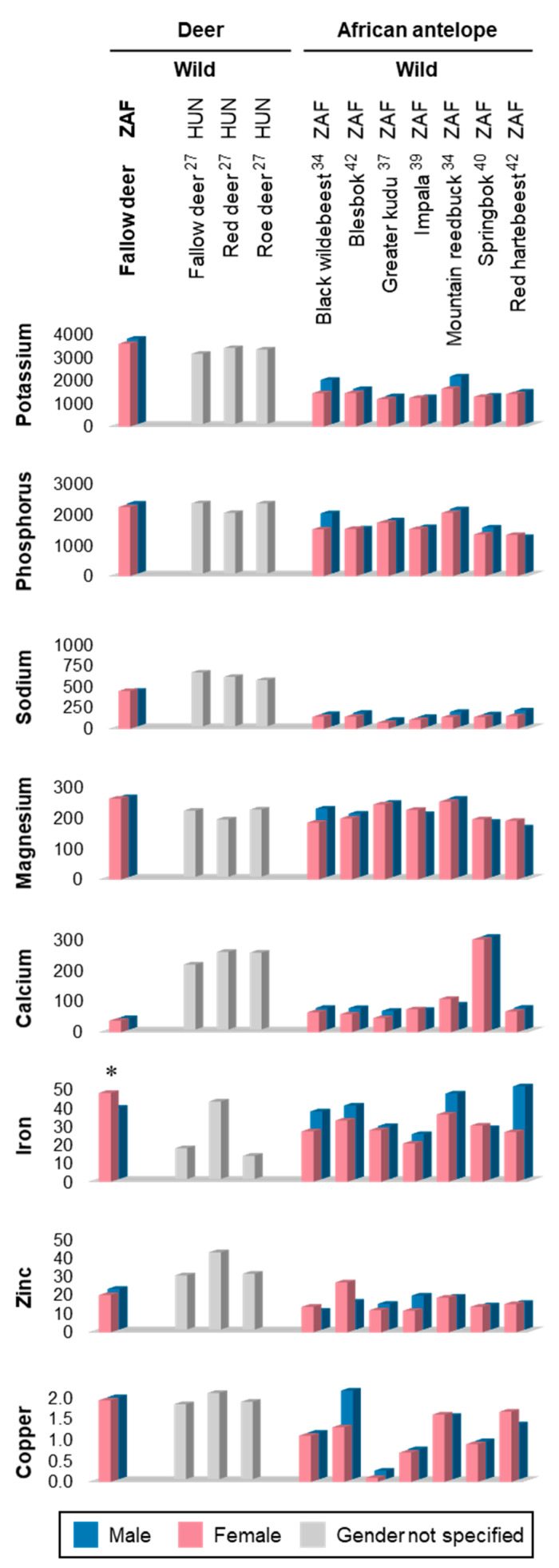
Mineral composition (mg/kg) of *longissimus thoracis et lumborum* muscles from selected game species, including sex comparisons where applicable. Values obtained in the present study appear in the first column (label marked in bold). The superscript numbers indicated after each species refer to references in the reference list [27,34,37,39,40,42]. * Indicates studies in which a significant sex effect was found. Abbreviations: HUN = Hungary; ZAF = South Africa.

Table 2 shows the mineral composition (mg/kg meat) of the LTL and BF from male and female fallow deer, whereas Table 3 compares the levels of selected elements in these muscles with relevant recommended dietary allowance (RDA) or adequate intake (AI) values, or with maximum intake (MI) levels in the case of potential contaminants. The mineral values in the fallow deer LTL are compared with those of various game species in Figure 2 (see also Supplementary Table S4).

The predominant macro-minerals determined in the fallow deer LTL and BF, in descending order of concentration, were potassium, phosphorus, sodium, and magnesium, with lower levels of calcium (Table 2). The primary essential micro-minerals measured in the muscles were iron and zinc, followed by copper and lower levels of manganese, selenium, and cobalt. The minerals detected that have undefined functions or can represent environmental contaminants included aluminium and lower levels of lead, strontium, and barium (Table 2). 

No significant interactions were observed between the main effects (sex × muscle) in terms of the mineral composition (Supplementary Table S3). When considering the effect of sex separately, significant differences were apparent only in the cases of the macro-mineral potassium and micro-minerals iron and selenium (Table 2). In contrast, the concentrations of seven of the 17 detected minerals were found to be significantly influenced by muscle (Table 2). The LTL contained significantly higher calcium concentrations compared with the BF, while the BF contained higher magnesium, phosphorus, and potassium concentrations. The LTL was further found to comprise higher concentrations of the essential micro-minerals iron and zinc, as well as the potential contaminant aluminium, relative to the BF. 

## 4. Discussion 

### 4.1. Proximate Composition

Broadly speaking, mammalian skeletal muscle is considered to comprise ca. 75% water, 19% protein, 1–10% lipid, 1% ash, and small amounts of other miscellaneous non-protein components [46,47]. In comparison, the concentrations of the proximate components in the six fallow deer muscles ranged from 73.3–76.2% moisture, 20.4–23.1% protein, 2.2–3.2% IMF, and 1.1–1.5% ash. 

The finding that sex did not influence the proximate composition of fallow deer muscles is in agreement with the sex comparison results reported for the same six muscles from common eland (*Taurotragus oryx*) harvested in South Africa, as well as with those reported for the LTL of greater kudu (*T*. *strepsiceros*), blesbok (*D*. *pygargus phillipsi*), and blue wildebeest (*Connochaetus taurinus*) from South Africa (Figure 1) [34,35,36,37]. Nonetheless, it is recognised that female ungulates tend to deposit more IMF than males [18] and such a trend has been documented in the LTL of impala (*A*. *melampus*) and springbok (*A*. *marsupialis*), as well as wild red deer (*Cervus elaphus*) and roe deer (*Capreolus capreolus*) [29,30,39,40]. On the other hand, higher levels of IMF have been recorded in the meat of male red hartebeest (*Alcelaphus buselaphus caama*) from South Africa and wild fallow deer (*D. dama*) bucks from Poland relative to their female counterparts (Figure 1), with no indications of the potential reasons for such differences [28,42]. Additionally, several studies have reported significant sex effects on the protein content of the LTL, with levels being higher in females in the case of mountain reedbuck (*Redunca fulvorufula*), black wildebeest (*Connochaetes gnou*), and roe deer and higher in males in the case of wild fallow deer (Figure 1) [28,30,34].

Significant muscle effects on proximate composition were anticipated in this study as skeletal muscles are known to differ in their function and activity levels, which is accordingly reflected by differences in their constituent muscle fibre composition, IMF levels, and connective tissue contents [47]. As further expected, the moisture contents of the fallow deer muscles varied considerably in line with fluctuating protein and IMF contents; however, the correlations between moisture and protein were stronger than those found between moisture and fat. Similar results have been reported for blesbok muscle and have been attributed to the low IMF content of game meat [48].

The result that the SM and LTL had the highest protein contents among the evaluated fallow deer muscles is in accordance with findings from studies on the same six muscles in eland and blue wildebeest [37,49]. Moreover, the protein to moisture ratios found for the various fallow deer muscles (range = 0.27:1 to 0.31:1) are higher than those typically associated with red meat (0.26:1 to 0.28:1) [16], suggesting a superior protein content in the former. Game meat and venison are indeed considered to be higher in protein than the meat from domestic livestock [27], and the protein content determined for the fallow deer LTL in this study accordingly surpasses those found for South African mutton, as well as grass- and grain-fed cattle (Figure 1) [43,44]. The present protein values nevertheless compare well with those measured in the LTL of farmed and wild fallow-, red- and roe-deer from Europe, while falling approximately midway in the range of protein values documented for indigenous African antelope species (ca. 19–26%; Figure 1). Wild fallow deer meat from South Africa therefore appears to represent a protein-dense meat source, with a 100 g portion being capable of contributing between 38–43% of the daily adult protein requirement [50], depending on the muscle consumed. 

Venison is generally reported to contain <3% IMF [15] and in this study, this value was exceeded only in the case of the BF. The higher IMF content of the BF might be partially explained by considering the muscle fibre composition and metabolic capacities of fallow deer muscles, at least in terms of comparisons with the SM and LTL for which such information exists. In a study on wild fallow deer from Poland, all three muscles (BF, SM, LTL) were found to predominantly comprise fast glycolytic (type IIB) muscle fibres, but the BF reportedly contained a higher percentage of slow oxidative (type I) fibres relative to the SM and LTL [51]. Since type I fibres are characterised by higher lipid concentrations than type IIB fibres [22], muscles showing higher proportions of the former could be expected to contain higher IMF levels. Nonetheless, it is not known whether the same patterns in muscle fibre composition occur in South African fallow deer and a strict correlation between fibre types and IMF contents may not be universally applicable to all genotypes and muscle types [17].

The IMF values measured here are lower than those found in the LTL of South African sheep, goat, and grain-fed cattle (Figure 1) [39,40,41]. While the present IMF values are comparable with those found in the LTL of wild fallow deer from Hungary (2.5%) [27], they are higher than those reported for wild and farmed fallow-, red-, and roe-deer from other parts of Europe, and also exceed those found for most indigenous antelope (Figure 1). It should nevertheless be highlighted that the fallow deer in this study were all harvested in winter and thus, a seasonal effect on the IMF contents cannot be ruled out. Seasonal changes in the body condition and meat composition of wild ungulates are linked to variations in rainfall patterns, vegetation availability, and feeding behaviours of the animals, which in turn influences their plane of nutrition [48,52]. Studies in Europe suggest that wild deer accumulate fat reserves in winter and indeed, significantly higher IMF values have been recorded in the meat from winter-harvested fallow deer in Poland relative to those from summer-harvested animals [52]. This said, the study area from which the present fallow deer were harvested is considered a non-seasonal rainfall region (i.e., rain all year round), implying that the animals should not be exposed to substantial differences in the quantity and quality of forage available to them across the seasons. Since the area contains a combination of C_3_ and C_4_ grass species, winter rainfalls are likely to favour the growth of the C_3_ grasses and summer rainfalls that of C_4_ species, and relatively good quality nutrition should consequently be available throughout the year [48]. 

The range of ash values determined in the fallow deer muscles are generally consistent with those found in the LTL of most deer and indigenous game animals, although lower ones have been reported in grain-fed cattle (Figure 1) [43].

### 4.2. Mineral Composition

Muscle tissue, particularly red meat, is regarded as an important source of essential macro-minerals, containing high levels of potassium and phosphorus, moderate levels of sodium and magnesium, but relatively low levels of calcium. It further represents a valuable source of essential micro-minerals (e.g., iron, copper, zinc, cobalt, manganese, selenium, molybdenum), many of which are exclusively present in muscle tissue or have higher bioavailability compared with those in plant tissues [53]. 

The macro-mineral concentrations determined in the fallow deer muscles followed a comparable pattern to that previously described for muscle tissue by Ortega-Barrales and Fernández-de Córdova [53]. However, the levels of potassium, phosphorus, and magnesium in the fallow deer muscles are higher than those summarised by the aforementioned authors for beef and lamb [53], whereas the levels of sodium and calcium are lower than in the latter. Compared with indigenous antelope and deer from Hungary, similar findings of higher potassium, phosphorus, and magnesium and lower calcium levels in South African fallow deer muscles can be observed (Figure 2). It is also notable that the present Na levels are higher than those found for indigenous antelope, while being lower than those reported for Hungarian deer species (Figure 2).

In terms of essential micro-minerals, the iron and copper concentrations determined in the fallow deer muscles are higher than those reported for beef and sheep [43,44], as well as being higher or comparable to those found in the meat of indigenous antelope and deer from Hungary (Figure 2). The present zinc levels are, however, lower than those recorded for beef and sheep [43,44], as well as Hungarian deer [27], although these are mostly higher than those reported for indigenous antelope (Figure 2). It should nevertheless be acknowledged that the techniques used for measuring mineral concentrations can differ widely between individual laboratories and this may potentially contribute to some of the variation observed between species for certain minerals.

It has previously been suggested that sex does not represent a determining factor in the levels of macro-minerals in meat products [53]. This is also reflected by various studies on wild deer and indigenous antelope species, where no sex differences have been reported in terms of the mineral compositions of the LTL (Figure 2). In this study, only the levels of potassium, iron, and selenium were found to be significantly influenced by sex. The differences in the potassium and iron content could potentially have been related to differences in the ages of the animals. The concentrations of potassium in muscle tissue are known to decrease with increasing age, while those of iron are reported to increase with increasing age [54]. As previously mentioned, high hunting pressure was observed to have reduced the number of large fallow deer buck in the study area during harvesting. This could have led to the harvested females being somewhat older than the males, potentially explaining the lower potassium and higher iron contents of the female muscles. 

Compared to the case of sex, considerably more variation was observed in the mineral concentrations between individual fallow deer muscles. Although the latter variations may be linked to differences in muscle fibre composition, such comparisons are limited in this study due to a lack of data on the muscle fibre profiles of local fallow deer. Nonetheless, muscles containing higher proportions of slow oxidative (type I) fibres might be expected to contain higher levels of calcium, iron, and zinc, but lower levels of potassium, than those muscles comprising mostly of fast glycolytic (type IIB) fibres [22,55].

In order to better relate the current mineral values to human health, the levels of selected elements in the fallow deer muscles were compared with relevant RDAs, AIs, and MI levels. From this comparison, it appears that the greatest contribution that a 100 g portion of fallow deer meat could make in meeting the RDA or AI requirements [56] would be through the supply of phosphorus, iron, copper, and zinc, and to a lesser extent, via the supply of potassium and magnesium (Table 3). In terms of potential contaminants in the meat, it is recognised that free-living animals are susceptible to the accumulation of certain metals/metalloids (especially cadmium, mercury, and lead) if they reside in anthropogenically polluted areas [57], which in turn can have adverse effects on human health. Nonetheless, neither cadmium nor mercury were detected in the fallow deer muscles in this study. Although high lead levels in game meat may also be due to contamination from lead ammunition (i.e., bullets) [57], it is clear that the lead concentrations measured in the fallow deer LTL and BF were well below the maximum levels specified for lead in meat (0.1 mg/kg) in South Africa [58] (Table 3). With respect to aluminium, a tolerable weekly intake (TWI) has been set for this element of 1 mg/kg body weight/week [59], which would equate to ca. 70 mg per week for a 70 kg individual. Although the aluminium levels measured in the fallow deer LTL and BF were relatively high compared to other potential contaminants, similar or higher levels (5–10 mg/kg) have been determined in bakery products, vegetables, seafood, dairy products, processed meats, tea, herbs, spices, and cocoa products [59].

## 5. Conclusions

This is the first study to evaluate the chemical meat quality of wild fallow deer in South Africa and in fact, in Africa more generally. Its novelty and importance also extend to the mineral analyses since there is currently little information on the mineral composition of wild ungulates, particularly game and deer. By comparing the present values with those from other game, deer, and livestock, a central source has also been generated through which a wealth of information can be accessed on the meat quality of different species. Furthermore, this study adds to a limited pool of knowledge on the chemical composition of alternative animal proteins, which is imperative as meat demand continues to escalate and prospects of augmenting livestock productivity is increasingly constrained.

Although some compositional differences were evident between the fallow deer muscles and to a lesser extent, sexes, comparison of the present results with relevant dietary guidelines and data for alternative game and domestic species indicates that fallow deer meat can be considered as a protein-dense foodstuff, with a low fat content and favourable mineral composition. Moreover, since all fallow deer in South Africa are currently wild, free roaming, and mostly unaffected by human interventions, their meat could prospectively be promoted as “free range” and “natural”. All indications are thus that fallow deer could serve as a healthy meat source and contribute to food security and economic revenue generation in South Africa, especially given that most fallow deer harvested in the country are surplus animals. Nonetheless, while this study has generated baseline data on the meat composition of local fallow deer, the meat industry may benefit from further work employing larger sample sizes and additionally assessing the potential effects of extrinsic factors (i.e., season, diet, slaughter age) on the evaluated chemical properties.

## Figures and Tables

**Table 1:** Mean (± standard error) proximate composition in g/100 g (%) of six different muscles from fallow deer (*n* = 12), as influenced by muscle and sex.

Parameter (g/100 g)	Muscle	Total Group ^†^	*p*-Value	Sex		*p*-Value
		(*n* = 12)	Muscle	Male (*n* = 6)	Female (*n* = 6)	Sex
**Moisture**	**LTL**	73.8 ^a^ ± 0.22	**<0.0001**	74.2 ± 0.36	73.4 ± 0.18	0.1187
	**BF**	74.5 ^b^ ± 0.20		74.8 ± 0.29	74.2 ± 0.23	
	**SM**	73.3 ^a^ ± 0.21		73.4 ± 0.25	73.3 ± 0.36	
	**ST**	75.8 ^c^ ± 0.28		75.5 ± 0.36	76.0 ± 0.44	
	**IS**	76.1 ^c^ ± 0.16		76.2 ± 0.19	75.9 ± 0.25	
	**SS**	76.2 ^c^ ± 0.16		76.6 ± 0.11	75.9 ± 0.22	
**Protein**	**LTL**	22.7 ^a^ ± 0.20	**<0.0001**	22.6 ± 0.35	22.7 ± 0.23	0.9636
	**BF**	21.9 ^b^ ± 0.24		21.8 ± 0.27	22.0 ± 0.43	
	**SM**	23.1 ^a^ ± 0.23		23.1 ± 0.30	23.0 ± 0.39	
	**ST**	20.9 ^cd^ ± 0.33		21.1 ± 0.50	20.6 ± 0.45	
	**IS**	21.3 ^c^ ± 0.15		21.3 ± 0.10	21.4 ± 0.30	
	**SS**	20.4 ^d^ ± 0.17		20.2 ± 0.24	20.6 ± 0.24	
**Lipid**	**LTL**	2.8 ^a^ ± 0.16	**<0.0001**	2.5 ± 0.09	3.0 ± 0.26	0.2967
	**BF**	3.2 ^b^ ± 0.20		3.4 ± 0.36	3.1 ± 0.19	
	**SM**	2.9 ^ab^ ± 0.10		3.0 ± 0.19	2.9 ± 0.11	
	**ST**	2.7 ^a^ ± 0.12		2.4 ± 0.11	2.9 ± 0.17	
	**IS**	2.2 ^c^ ± 0.08		2.1 ± 0.06	2.3 ± 0.14	
	**SS**	2.8 ^a^ ± 0.15		2.8 ± 0.22	2.9 ± 0.22	
**Ash**	**LTL**	1.1 ^a^ ± 0.02	**0.0003**	1.1 ± 0.02	1.1 ± 0.03	0.7267
	**BF**	1.2 ^a^ ± 0.03		1.2 ± 0.05	1.2 ± 0.03	
	**SM**	1.4 ^bc^ ± 0.08		1.3 ± 0.06	1.5 ± 0.13	
	**ST**	1.5 ^c^ ± 0.07		1.4 ± 0.07	1.5 ± 0.12	
	**IS**	1.3 ^ab^ ± 0.05		1.3 ± 0.07	1.2 ± 0.08	
	**SS**	1.4 ^bc^ ± 0.06		1.4 ± 0.10	1.4 ± 0.09	

LTL = longissimus et thoracis lumborum; BF = biceps femoris; SM = semimembranosus; ST = semitendinosus; IS = infraspinatus; SS = supraspinatus; **^†^** Total group: different superscripts within a column for a specific parameter indicate significant differences (*p* ≤ 0.05) among individual muscles.

**Table 2:** Mean (±standard error) mineral composition (mg/kg meat) of fallow deer (*n* = 12) *longissimus thoracis et lumborum* and *biceps femoris*, as influenced by muscle and sex. Dashed lines = not detected.

Mineral (mg/kg Meat)	Total Group ^†^			Sex ^#^				
	(*n* = 12)		*p*-Value	Male (*n* = 6)		Female (*n* = 6)		*p*-Value
	LTL	BF	Muscle	LTL	BF	LTL	BF	Sex
***Macro-minerals***								
Potassium (K)	3622.478 ± 33.775 *	**3743.060 ± 50.492**	**0.0004**	**3670.861 ^a^ ± 34.042**	**3852.473 ^b^ ± 45.258**	**3574.096 ^a^ ± 54.076**	**3633.647 ^a^ ± 66.186**	**0.0446**
Phosphorus (P)	**2245.846 ± 18.126**	**2301.909 ± 20.857**	**0.0208**	2256.688 ± 26.579	2313.660 ± 26.152	2235.003 ± 26.308	2290.158 ± 34.278	0.5307
Sodium (Na)	435.319 ± 8.105	432.910 ± 9.621	0.7496	419.832 ± 11.758	426.394 ± 16.299	450.806 ± 7.408	439.427 ± 11.165	0.1841
Magnesium (Mg)	**259.402 ± 1.353**	**272.992 ± 2.072**	**0.0001**	256.985 ± 1.758	271.213 ± 2.261	261.818 ± 1.621	274.771 ± 3.536	0.1609
Calcium (Ca)	**37.143 ± 0.720**	**34.810 ± 0.656**	**0.0168**	36.809 ± 1.190	34.793 ± 1.109	37.478 ± 0.905	34.826 ± 0.817	0.7733
***Micro-minerals***								
Iron (Fe)	**43.196 ± 1.954**	**38.294 ± 1.352**	**0.0023**	**38.414 ^ab^ ± 1.811**	**34.788 ^a^ ± 1.045**	**47.978 ^c^ ± 2.093**	**41.800 ^b^ ± 1.426**	**0.0019**
Zinc (Zn)	**20.844 ± 0.630**	**14.955 ± 0.499**	**0.0000**	21.700 ± 0.843	14.521 ± 0.481	19.988 ± 0.862	15.390 ± 0.888	0.6644
Silicon (Si)	6.233 ± 0.316	5.506 ± 0.345	0.1599	6.522 ± 0.507	5.686 ± 0.669	5.944 ± 0.388	5.325 ± 0.254	0.3500
Copper (Cu)	1.942 ± 0.071	2.014 ± 0.055	0.1181	1.944 ± 0.132	2.008 ± 0.089	1.939 ± 0.069	2.020 ± 0.074	0.9762
Manganese (Mn)	0.201 ± 0.008	0.218 ± 0.008	0.0618	0.202 ± 0.012	0.203 ± 0.008	0.200 ± 0.013	0.233 ± 0.010	0.3038
Selenium (Se)	0.146 ± 0.007	**0.139 ± 0.007**	**0.3232**	**0.139 ^ab^ ± 0.011**	**0.122 ^a^ ± 0.004**	**0.153 ^b^ ± 0.007**	**0.155 ^b^ ± 0.008**	**0.0208**
Chromium (Cr)	0.058 ± 0.019	0.071 ± 0.028	0.7038	0.037 ± 0.015	0.104 ± 0.053	0.078 ± 0.034	0.037 ± 0.016	0.7043
Cobalt (Co)	0.003 ± 0.001	0.002 ± 0.000	0.3357	0.004 ± 0.002	0.001 ± 0.001	0.002 ± 0.000	0.002 ± 0.001	0.4963
Molybdenum (Mo)	---	---		---	---	---	---	
***Undefined functions or environmental contaminants***								
Aluminium (Al)	**5.763 ± 0.379**	**3.170 ± 0.55**	**0.0033**	5.516 ± 0.585	2.696 ± 0.913	6.010 ± 0.514	3.644 ± 0.623	0.3111
Lead (Pb)	0.044 ± 0.029	0.009 ± 0.006	0.3958	0.011 ± 0.004	0.014 ± 0.011	0.020 ± 0.011	0.003 ± 0.003	0.3729
Strontium (Sr)	0.019 ± 0.003	0.014 ± 0.002	0.2003	0.024 ± 0.005	0.017 ± 0.004	0.014 ± 0.003	0.013 ± 0.002	0.0586
Barium (Ba)	0.014 ± 0.002	0.010 ± 0.001	0.0727	0.018 ± 0.003	0.010 ± 0.002	0.011 ± 0.002	0.009 ± 0.001	0.1048
Antimony (Sb)	---	---		---	---	---	---	
Arsenic (As)	---	---		---	---	---	---	
Boron (B)	---	---		---	---	---	---	
Cadmium (Cd)	---	---		---	---	---	---	
Mercury (Hg)	---	---		---	---	---	---	
Nickel (Ni)	---	---		---	---	---	---	
Titanium (Ti)	---	---		---	---	---	---	
Vanadium (V)	---	---		---	---	---	---	

LTL = *longissimus et thoracis lumborum;* BF = *biceps femoris*. **^†^** Total group: bold text across the rows indicates significant differences between muscles. **^#^** Sex: different superscripts across individual rows indicates significant (*p* ≤ 0.05) differences between males and females. * The bold text indicates significant differences in values across the rows.

**Table 3:** Mean mineral levels (mg/kg meat) found in fallow deer (*n* = 12) *longissimus thoracis et lumborum* and *biceps femoris* muscles compared with relevant dietary recommendations or maximum intake levels for adults. The superscript numbers indicated for the RDA/AI and maximum levels refer to references in the reference list.

Element	LTL (mg/kg)	BF (mg/kg)	RDA/AI ^56^ (mg/day)	RDA/AI met by 100 g LTL	RDA/AI met by 100 g BF	Maximum Level
*Essential Macro- and Micro-Minerals*						
Calcium (Ca)	37.143	34.810	1000–1200	0.31–0.37%	0.29–0.35%	---
Copper (Cu)	1.942	2.014	0.9	21.6%	22.4%	---
Iron (Fe)	43.196	38.294	8–18	24–54%	21.3–47.9%	---
Magnesium (Mg)	259.402	272.992	310–400	6.5–8.4%	6.8–8.8%	---
Manganese (Mn)	0.201	0.218	1.8–2.3	0.9–1.1%	0.9–1.1%	---
Potassium (K)	3622.478	3743.060	4700	7.7%	8.0%	---
Phosphorus (P)	2245.846	2301.909	700	32.1%	32.9%	---
Sodium (Na)	435.319	432.910	1500	2.9%	2.9%	---
Zinc (Zn)	20.844	14.955	8–11	18.9–26.1%	13.6–18.7%	---
***Elements with undefined functions or environmental contaminants***						
Lead (Pb)	0.044	0.009	---	---	---	0.1 mg/kg (meat) ^58^
Aluminium (Al)	5.763	3.170	---	---	---	1 mg/kg bw/week ^59^

LTL = *longissimus et thoracis lumborum;* BF = *biceps femoris*; RDA = recommended dietary allowance; AI = adequate intake; dashed lines = not determined; bw = body weight.

## Supplementary Materials

The following are available online at https://www.mdpi.com/2304-8158/9/5/598/s1, Table S1 Carcass characteristics, individual muscle weights (right-hand side), and ultimate pH values of muscles from male (*n* = 6) and female (*n* = 6) fallow deer.; Table S2 Proximate composition of *longissimus thoracis et lumborum* muscles from selected game and domestic livestock species, including sex comparisons where applicable. Values obtained in the present study appear in the first row. Significant differences are indicated in bold. NS = not specified; Table S3 Interactions between the main effects (sex × muscle) in the measurement of the proximate and mineral composition of wild fallow deer (*n* = 12) from South Africa. All interactions were non-significant; Table S4 Mineral composition (mg/kg) of *longissimus thoracis et lumborum* muscles from selected game species, including sex comparisons where applicable. Values obtained in the present study appear in the first row. Significant differences are indicated in bold. NS = not specified.
