# Supplementary material for: Chemical Composition of Wild Fallow Deer (Dama Dama) Meat from South Africa: A Preliminary Evaluation
Source: Foods. 2020 May 7;9(5):598. doi: 10.3390/foods9050598 (PMC7278756; doi:10.3390/foods9050598)
Supplement: Supplementary file 1 [file foods-09-00598-s001.pdf]

SUPPLEMENTARY MATERIAL

Table S1 Carcass characteristics, individual muscle weights (right-hand side) and ultimate pH values of muscles from male (n = 6) and female (n = 6) fallow deer.

Table S2 Proximate composition of *longissimus thoracis et lumborum* muscles from selected game and ruminant livestock species, including sex comparisons where applicable. Values obtained in the present study appear in the first row. Significant differences are indicated in bold. NS = not specified.

Table S3 Interactions between the main effects (sex × muscle) in the measurement of the proximate and mineral composition of wild fallow deer (n = 12) from South Africa. All interactions were non-significant.

Table S4 Mineral composition (mg/kg) of *longissimus thoracis et lumborum* muscles from selected game species, including sex comparisons where applicable. Values obtained in the present study appear in the first row. Significant differences are indicated in bold. NS = not specified.

Table S1 Carcass characteristics, individual muscle weights (right-hand side) and ultimate pH values† of muscles from male (n = 6) and female (n = 6) fallow deer

		Carcass characteristics						Muscle weights						Ultimate pH (36 h)					
**Sex**	**No.**	**Slaughter weight (kg)**	**Warm carcass weight (kg)**	**Cold carcass weight (kg)**	**Dressing percentage (%)**	**Kidney fat (kg)**	**Gut fat (kg)**	**LTL (kg)**	**BF (kg)**	**SM (kg)**	**SS (kg)**	**IS (kg)**	**ST (kg)**	**LTL**	**BF**	**SM**	**SS**	**IS**	**ST**
**Males (n = 6)**	1	53.5	32.7	32.2	60.2	0.037	0.048	1.154	0.967	0.822	0.207	0.215	0.250	5.30	5.48	5.34	5.66	5.67	5.47
	2	49.6	32.1	31.4	63.3	0.060	0.066	1.100	0.973	0.787	0.107	0.101	0.236	5.55	5.41	5.59	5.52	5.43	5.40
	3	41.2	24.0	23.1	56.1	0.023	0.072	0.937	0.742	0.576	0.162	0.165	0.180	5.66	5.40	5.43	5.56	5.56	5.41
	4	39.5	24.5	24.0	60.8	0.091	0.089	0.901	0.705	0.620	0.147	0.156	0.179	5.57	5.41	5.51	5.70	5.73	5.51
	5	39.5	23.8	23.3	59.0	0.056	0.152	0.847	0.724	0.618	0.136	0.179	0.185	5.34	5.62	5.58	5.62	5.60	5.60
	6	37.8	23.8	23.4	61.9	0.193	0.121	0.888	0.710	0.619	0.143	0.171	0.178	5.63	5.74	5.54	5.71	5.81	5.40
	Mean	43.5	26.8	26.2	60.2%	0.077	0.091	0.971	0.803	0.673	0.150	0.164	0.201	5.51	5.51	5.50	5.63	5.63	5.47
	SE	2.63	1.77	1.77	0.01	0.025	0.016	0.051	0.053	0.042	0.014	0.015	0.013	0.062	0.057	0.039	0.031	0.055	0.033
**Females (n = 6)**	2	51.4	29.8	29.2	56.9	0.140	0.736	0.948	0.985	0.708	0.174	0.213	0.228	5.13	5.33	5.37	5.42	5.50	5.24
	1	49.7	31.1	30.6	61.6	0.283	0.691	1.104	0.884	0.770	0.200	0.270	0.228	5.47	5.55	5.50	5.81	5.78	5.43
	3	45.0	28.0	27.6	61.3	0.153	0.750	1.153	0.915	0.736	0.178	0.196	0.222	5.38	5.43	5.40	5.46	5.48	5.42
	5	44.9	27.3	26.9	60.0	0.109	0.566	1.014	0.890	0.704	0.170	0.192	0.215	5.23	5.42	5.39	5.39	5.52	5.26
	4	35.2	22.2	21.9	62.2	0.040	0.196	0.921	0.658	0.602	0.132	0.153	0.182	5.44	5.47	5.21	5.43	5.55	5.51
	6	31.9	19.3	19.0	59.6	0.052	0.094	0.854	0.627	0.543	0.120	0.120	0.166	5.46	5.48	5.45	5.58	5.49	5.48
	Mean	43.0	26.3	25.9	60.3	0.130	0.506	0.999	0.826	0.677	0.162	0.190	0.206	5.35	5.45	5.39	5.52	5.55	5.39
	SE	3.19	1.86	1.83	0.01	0.036	0.118	0.046	0.060	0.035	0.012	0.021	0.011	0.057	0.030	0.040	0.065	0.046	0.046

Abbreviations: LTL = *longissimus et thoracis lumborum;* BF = *biceps femoris*; SM = *semimembranosus*; ST = *semitendinosus*; IS = *infraspinatus*; SS = *supraspinatus*; SD = standard deviation; SE = standard error.

† pH values measured at 36-hours post mortem

Table S2 Proximate composition of *longissimus thoracis et lumborum* muscles from selected game and domestic livestock species, including sex comparisons where applicable. Values obtained in the present study appear in the first row. Significant differences are indicated in bold. NS = not specified.

Common name	Species	Country	Age	Sex	N	Production	Moisture (%)	Protein (%)	Fat (%)	Ash (%)	Reference
DEER											
Fallow deer	*Dama dama*	South Africa	NS	Female<br>Male	6<br>6	Wild<br>Wild	73.4<br>74.2	22.7<br>22.6	3.0<br>2.5	1.1<br>1.1	THIS STUDY
Fallow deer	*Dama dama*	Hungary	10 months	NS	10	Wild	74.90	22.00	2.50	1.08	Zomborszky et al., 1996
Fallow deer	*Dama dama*	Poland	17-18 months	Female<br>Male	10<br>11	Wild<br>Wild	**75.10**<br>**74.29**	**21.76**<br>**22.79**	**0.30**<br>**0.50**	1.07<br>1.10	Piaskowska et al., 2015
Fallow deer	*Dama dama*	Poland	18 months	Male	6	Farmed	74.33	22.46	0.24	1.09	Daszkiewicz et al., 2015
Fallow deer	*Dama dama*	Czech Republic	2.5 years	Female<br>Male	9<br>9	Farmed<br>Farmed	76.48<br>73.77	21.86<br>22.4	1.65<br>1.85	1.19<br>1.21	Švrčula et al., 2019
Red deer	*Cervus elaphus*	Poland	4-6 years	Female<br>Male	23<br>19	Wild<br>Wild	**74.43**<br>**75.22**	22.41<br>22.01	**0.96**<br>**0.56**	1.09<br>1.10	Daszkiewicz et al., 2009
Red deer	*Cervus elaphus*	Czech Republic	16 months	Male	9	Farmed	74.27	22.14	0.81		Bureš et al, 2015
Roe deer	*Capreolus capreolus*	Poland	3-4 years	Female<br>Male	25<br>16	Wild<br>Wild	**73.80**<br>**75.32**	**22.79**<br>**21.84**	**1.46**<br>**0.83**	1.12<br>1.13	Daszkiewicz et al., 2012
ANTELOPE											
Black wildebeest	*Connochaetes gnou*	South Africa	Adult, sub-adult	Female<br>Male	12<br>7	Wild<br>Wild	75.21<br>74.69	**20.73**<br>**19.42**	1.13<br>0.97	1.25<br>1.29	Van Schalkwyk, 2004
Blue wildebeest	*Connochaetus taurinus*	South Africa	Adults, sub-adults, calves	Female<br>Male	10<br>5	Wild<br>Wild	75.99<br>75.55	22.83<br>23.31	1.38<br>1.26	1.38<br>1.26	Van Schalkwyk, 2004
Common eland	*Taurotragus oryx*	South Africa	2-6 years	Female<br>Male	6<br>6	Wild<br>Wild	76.6<br>77.1	21.6<br>21.2	1.48<br>1.45	1.1<br>1.1	Needham et al., 2019
Blesbok	*Damaliscus pygargus phillipsi*	South Africa	Adult	Female<br>Male	37<br>28	Wild<br>Wild	74.88-75.33	22.18-22.45	0.21-1.48	1.24-1.38	Hoffman et al., 2008
Greater kudu	*Tragelaphus strepsiceros*	South Africa	NS	Female<br>Male	10<br>8	Wild<br>Wild	74.14<br>74.49	24.3<br>23.6	1.56<br>1.58	1.29<br>1.23	Mostert & Hoffman, 2007
Common duiker	*Sylvicapra grimmia*	South Africa	>26 months	Male	10	Wild	71.41	25.71	2.12	1.29	Hoffman & Ferreira, 2004

Impala	*Aepyceros melampus*	South Africa	Adult	Female	7	Wild	74.01	23.07	**2.4**	1.16	Hoffman et al., 2009
				Male	11	Wild	74.96	22.63	**2.06**	1.22	
Mountain reedbuck	*Redunca fulvorufula*	South Africa	Adult Non-trophy	Female	19	Wild	72.59	**24.51**	2.43	1.22	Van Schalkwyk, 2004
				Male	10	Wild	72.76	**23.68**	2.94	1.23	
Springbok	*Antidorcas marsupialis*	South Africa	Adult, sub-adult	Female	76	Wild	**73.39**	18.8-21.16	**3.13**	1.28	Hoffman et al., 2007
				Male	90	Wild	**74.24**		**1.35**	1.24	
Springbok	*Antidorcas marsupialis*	South Africa	>2 years	Both	27	Wild	73.8	22.06	3.07	1.14	Neethling et al., 2018
Red hartebeest	*Alcelaphus buselaphus caama*	South Africa	Adult, sub-adult	Female	27	Wild	74.75	23.1	**2.81**	1.22	Smit, 2004
				Male	21	Wild	75.08	23.34	**4.69**	1.16	
DOMESTIC SPECIES											
Cattle - grass fed (Bonsmara)	*Bos taurus*	South Africa	3-4 incisors	Male	20	Farmed	75.8	21.3	1.65	1.21	Moholisa et al., 2018
Cattle - grain fed (Bonsmara)	*Bos taurus*	South Africa	9-11 months	Male	20	Farmed	74.8	21.1	3.12	0.97	
Mutton (Dorper/merino – Age C, fat class 2)	*Ovis aries*	South Africa	>6 incisors	NS	18	Farmed	73.71	20.39	4.96	1.18	Sainsbury, 2009
Goat, indigenous	*Capra aegagrus hircus*	South Africa	2-6 teeth	Female	15	Farmed		23.52	4.03		Simela, 2005
				Male (castrate)	15	Farmed		23.76	4.07		

Table S3 Interactions between the main effects (sex × muscle) in the measurement of proximate and mineral composition of wild fallow deer (n = 12) from South Africa. All interactions were non-significant.

		**p-value**
		**sex*muscle**
Proximate components		
Moisture	g/100g	0.2114
Protein	g/100g	0.6676
Fat	g/100g	0.1387
Ash	g/100g	0.2213
Macro-minerals		
Potassium (K)	mg/kg	0.0677
Phosphorus (P)	mg/kg	0.9768
Sodium (Na)	mg/kg	0.4150
Magnesium (Mg)	mg/kg	0.9518
Calcium (Ca)	mg/kg	0.7128
Micro-minerals		
Iron (Fe)	mg/kg	0.4945
Zinc (Zn)	mg/kg	0.1712
Silicon (Si)	mg/kg	0.7156
Copper (Cu)	mg/kg	0.9558
Manganese (Mn)	mg/kg	0.1413
Selenium (Se)	mg/kg	0.4210
Chromium (Cr)	mg/kg	0.2592
Cobalt (Co)	mg/kg	0.2403
Molybdenum (Mo)	mg/kg	Not detected
Undefined functions or environmental contaminants		
Aluminium (Al)	mg/kg	0.4785
Lead (Pb)	mg/kg	0.0859
Strontium (Sr)	mg/kg	0.8330
Barium (Ba)	mg/kg	0.3430
Antimony (Sb)	mg/kg	Not detected
Arsenic (As)	mg/kg	Not detected
Boron (B)	mg/kg	Not detected
Cadmium (Cd)	mg/kg	Not detected
Mercury (Hg)	mg/kg	Not detected
Nickel (Ni)	mg/kg	Not detected
Titanium (Ti)	mg/kg	Not detected
Vanadium (V)	mg/kg	Not detected

Table S4 Mineral composition (mg/kg) of *longissimus thoracis et lumborum* muscles from selected game species, including sex comparisons where applicable. Values obtained in the present study appear in the first row. Significant differences are indicated in bold. NS = not specified.

Common name	Species	Country	Sex	N	Production	Potassium (mg/kg)	Phosphorus (mg/kg)	Sodium (mg/kg)	Magnesium (mg/kg)	Calcium (mg/kg)	Iron (mg/kg)	Zinc (mg/kg)	Copper (mg/kg)	Reference
**DEER**														
Fallow deer	*Dama dama*	South Africa	Female	6	Wild	3574.1	2235.0	450.8	261.8	37.5	**47.978**	19.988	1.939	THIS STUDY
			Male	6	Wild	3670.9	2256.7	419.8	257.0	36.8	**38.414**	21.7	1.944	
Fallow deer	*Dama dama*	Hungary	NS	10	Wild	3019.5	2269.0	635.0	213.6	210.1	16.56	28.89	1.78	Zomborszky et al., 1996
Red deer	*Cervus elaphus*	Hungary	NS	10	Wild	3268.7	1954.3	584.4	185.5	250.9	41.77	41.21	2.04	
Roe deer	*Capreolus capreolus*	Hungary	NS	10	Wild	3205.4	2263.0	546.8	217.2	248.5	12.48	29.76	1.83	
**ANTELOPE**														
Black wildebeest	*Connochaetes gnou*	South Africa	Female	12	Wild	1445.4	1514.3	144.8	184.3	64.7	27.3	13.7	1.1	Van Schalkwyk, 2004
			Male	7	Wild	1897.2	1944.3	144.8	220.4	69.8	36.4	10	1.1	
Blesbok	*Damaliscus dorcas phillipsi*	South Africa	Female	40	Wild	1449.8	1528.1	144.7	197.7	58.5	33.1	26.7	1.3	Smit, 2004
			Male	29	Wild	1504.7	1453.1	158.2	204.0	69.4	39.6	14.8	2.7	
Greater kudu	*Tragelaphus strepsiceros*	South Africa	Female	10	Wild	1190.0	1730.0	74.1	243.0	46.2	27.9	11.9	0.1	Mostert & Hoffman, 2007
			Male	8	Wild	1200.0	1720.0	75.9	239.0	60.1	28.5	13.7	0.2	
Impala	*Aepyceros melampus*	South Africa	Female	7	Wild	1242.8	1524.2	109.1	225.5	74.5	20.7	11.6	0.7	Hoffman et al., 2009
			Male	11	Wild	1159.5	1497.0	109.5	202.7	63.2	24.2	18.1	0.71	
Mountain reedbuck	*Redunca fulvorufula*	South Africa	Female	7	Wild	1640.0	2048.0	139.1	251.7	107.9	36.4	18.6	1.6	Van Schalkwyk, 2004
			Male	9	Wild	2046.0	2077.7	169.3	252.5	80.4	46.1	17.5	1.5	
Springbok	*Antidorcas marsupialis*	South Africa	Female	10	Wild	1286.5	1353.9	140.4	195.1	437.7	30.4	13.7	0.9	Hoffman et al., 2007
			Male	12	Wild	1217.5	1485.5	141.2	177.7	792.4	27.4	12.9	0.9	
Red hartebeest	*Alcelaphus buselaphus caama*	South Africa	Female	27	Wild	1409.6	1336.5	153.2	190.2	67.4	26.9	15.13	1.67	Smit, 2004
			Male	21	Wild	1392.2	1178.1	187.4	159.6	70.3	115.1	14.2	1.3	

References

Bureš, D.; Bartoň, L.; Kotrba, R.; Hakl, J. Quality attributes and composition of meat from red deer (*Cervus elaphus*), fallow deer (*Dama dama*) and Aberdeen Angus and Holstein cattle (*Bos taurus*). *J. Sci. Food. Agric.* **2015**, *95*, 2299–2306.

Daszkiewicz, T.; Hnatyk, N.; Dąbrowski, D.; Janiszewski, P.; Gugołek, A.; Kubiak, D.; Śmiecińskaa, K; Winarskia, R; Koba-Kowalczyka, M. A comparison of the quality of the *Longissimus lumborum* muscle from wild and farm-raised fallow deer (*Dama dama*). *Small Rum. Res.* **2015**, *129*, 77–83.

Daszkiewicz, T., Janiszewski, P., Wajda, S. Quality characteristics of meat from wild red deer (*Cervus elaphus* L.) hinds and stags. *J. Muscle Foods* **2009**, *20*, 428–448.

Daszkiewicz, T.; Kubiak, D.; Winarski, R.; Koba-Kowalczyk, M. The effect of gender on the quality of roe deer (*Capreolus capreolus* L.) meat. *Small Rum. Res.* **2012**, *103*, 169–175.

Hoffman, L.C.; Ferreira, A.V. Chemical composition of two muscles of the common duiker (*Sylvicapra grimmia*). *J. Sci. Food Agric.* **2004**, *84*, 1541–1544.

Hoffman, L.C.; Kroucamp, M.; Manley M. Meat quality characteristics of springbok (*Antidorcas marsupialis*). 2: Chemical composition of springbok meat as influenced by age, gender and production region. *Meat Sci.* **2007**, *76*, 762–767.

Hoffman, L.C.; Mostert, A.C.; Kidd, M.; Laubscher, L.L. Meat quality of kudu (*Tragelaphus strepsiceros*) and impala (*Aepyceros melampus*): Carcass yield, physical quality and chemical composition of kudu and impala *Longissimus dorsi* muscle as affected by gender and age. *Meat Sci.* **2009**, *83*, 788–795.

Hoffman, L.C.; Smit, K.; Muller, N. Chemical characteristics of blesbok (*Damaliscus dorcas phillipsi*) meat. *J. Food Compos. Anal.* **2008**, *21*, 315–319.

Moholisa, E.; Strydom, P.E.; Hugo, A. The effect of beef production system on proximate composition and fatty acid profile of three beef muscles. *S. Afr. J. Anim. Sci.* **2018**, *48*, 295–306.

Mostert, R.; Hoffman, L.C. Effect of gender on the meat quality characteristics and chemical composition of kudu (*Tragelaphus strepsiceros*), an African antelope species. *Food Chem.* **2007**, *104*, 565–570.

Needham, T.; Laubser, J.G.; Kotrba, R.; Bureš, D.; Hoffman, L.C. Sex influence on muscle yield and physiochemical characteristics of common eland (*Taurotragus oryx*) meat. *Meat Sci.* **2019**, *152*, 41–48.

Neethling, J.; Muller, M.; van der Rijst, M.; Hoffman, L.C. Sensory quality and fatty acid content of springbok (*Antidorcas marsupialis*) meat: Influence of farm location and sex. *J. Sci. Food Agric.* **2018**, *98*, 2548–2556.

Piaskowska, N.; Daszkiewicz, T.; Kubiak, D.; Janiszewski, P. The effect of gender on meat (*longissimus lumborum* muscle) quality characteristics in the fallow deer *Dama dama* L. *Ital. J. Anim. Sci.* **2015**, *14*, 3845, doi: 10.4081/ijas.2015.3845.

Sainsbury, J. Nutrient content and carcass composition of South African mutton with a focus on bioavailability of selected nutrients. MSc Thesis, University of Pretoria, Pretoria, South Africa, December 2009.

Simela, L. Meat characteristics and acceptability of chevon from South African indigenous goats. PhD Thesis, University of Pretoria, Pretoria, South Africa, June 2005.

Smit, K. Meat quality characteristics of blesbok (*Damaliscus dorcas phillipsi*) and red hartebeest (*Alcelaphus buselaphus caama*) meat. MSc Thesis, Stellenbosch University, Stellenbosch, South Africa, December 2004.

Švrčula, V.; Košinová, K.; Okrouhlá, M.; Chodová, D.; Hart, V. The effect of sex on meat quality of fallow deer (*Dama dama*) from the farm located in the Middle Bohemia. *Ital. J. Anim. Sci.* **2019**, *18*, 498–504.

Van Schalkwyk, S. Meat quality characteristics of three South African game species: Black wildebeest (*Connochaetes gnou*), blue wildebeest (*Connochaetes taurinus*), mountain reedbuck (*Redunca fulvorufula*). MSc Thesis, Stellenbosch University, Stellenbosch, South Africa, December 2004.

Zomborszky, Z.; Szentmihalyi, G.; Sarudi, I.; Horn, P.; Szabo, C.S. Nutrient composition of muscles in deer and boar. *J. Food Sci.* **1996**, *61*, 625–627.
